# What’s new in surgery – essentials 2023: the ISS young surgeon issue

**DOI:** 10.1515/iss-2024-0009

**Published:** 2024-03-07

**Authors:** Juliane Kröplin

**Affiliations:** Klinik und Poliklinik für Mund-, Kiefer- und Plastische Gesichtschirurgie, Universitätsmedizin Rostock, Schillingallee 35, 18057 Rostock, Germany

Dear colleagues,

“I am one of those who think like Nobel, that humanity will draw more good than evil from new discoveries.”

This famous quote by Marie Curie describes both the conviction and motivation behind the conception of this new Innovative Surgical Science issue you find here, and is intended to achieve one thing above all: constant anticipation of all the articles about the latest discoveries in surgery that will appear here for you in the future.

## Purpose

Surgery is in a state of flux. This affects not only scientific progress, but also us as a profession. In order to pass on to future generations the competence, drive, critical curiosity and continuous scientific development that characterize us and our profession, we have developed this concept:

Taking into account the most current literature, young specialists from the various surgical disciplines will present to you the developments and highlights of their individual surgical specialties.

## Methods

The fact that young surgery is taken into account in the specialized surgical journals is not new and very welcome. But what makes our issue special is nothing less than one of the basic responsibilities of the German Society of Surgery: “Scientific information for all surgeons”. We would like to introduce you to what else makes us special. We are:

1. Brand new:

Admittedly, the idea is not entirely new. It is based on the successful German-written book “What’s new in surgery” which was, after more than 20 years, last published in 2022 in cooperation between the German Society of Surgery and the professional Board of Surgery of Germany (BDC) [1]. *Brand new*, however, continues to be the literature and related scientific knowledge on which the present articles are based.

2. Interdisciplinary:

Interdisciplinary cooperation is a very special concern for us surgeons. Combining synergies and using them in our daily work for our patients gives us the necessary skills for the daily challenges of our great profession. This strength is now also reflected on a scientific level in this surgical interdisciplinary journal issue.

3. Digital:

Enabling access to knowledge flexibly in terms of time and place is one of the core competencies of digital knowledge formats. In addition, the aspect of sustainability and the conscious use of resource-saving media also play an important role.

4. Competent:

We are proud of Innovative Surgical Science’s high scientific standards. That is why we also support our junior authors through our peer review processes.

5. Open access:

According to current knowledge, open access publications are likely to be cited more frequently than conventional publications and attract the attention of the scientific public to a large extent [2], [3], [4]. Unrestricted access to scientific knowledge and the unimpeded flow of knowledge are also essential prerequisites for any research activity [5].

## Conclusion

The first issue of “What’s new in surgery – Essential 2023” offers all surgeons an overview of current trends and new evidence-based strategies – both concerning their own specialty – and beyond the surgical horizon. We hope you enjoy reading it.
